# miRNA array screening reveals cooperative MGMT-regulation between miR-181d-5p and miR-409-3p in glioblastoma

**DOI:** 10.18632/oncotarget.8618

**Published:** 2016-04-05

**Authors:** Susanna Khalil, Enrica Fabbri, Alessandra Santangelo, Valentino Bezzerri, Cinzia Cantù, Gianfranco Di Gennaro, Alessia Finotti, Claudio Ghimenton, Albino Eccher, Maria Dechecchi, Aldo Scarpa, Brian Hirshman, Clark Chen, Manuela Ferracin, Massimo Negrini, Roberto Gambari, Giulio Cabrini

**Affiliations:** ^1^ Department of Pathology and Diagnostics, Laboratory of Molecular Pathology, University Hospital, Verona, Italy; ^2^ Section of Pathology and Histology, University Hospital, Verona, Italy; ^3^ Department of Life Sciences and Biotechnology, Section of Biochemistry and Molecular Biology, University of Ferrara, Ferrara, Italy; ^4^ Department of Morphology, Surgery and Experimental Medicine and Laboratory for Technologies of Advanced Therapies (LTTA), University of Ferrara, Ferrara, Italy; ^5^ Applied Research on Cancer Network (ARC-NET), University and Hospital Trust, Verona, Italy; ^6^ Center for Theoretical and Applied Neuro-oncology, Moores Cancer Center, Department of Neurosurgery, University of California San Diego, San Diego, CA, USA

**Keywords:** glioblastoma, MGMT, miR-409-3p, miR-181d-5p

## Abstract

The levels of expression of O^6^-methylguanine-DNA methyltransferase (MGMT) are relevant in predicting the response to the alkylating chemotherapy in patients affected by glioblastoma. MGMT promoter methylation and the published MGMT regulating microRNAs (miRNAs) do not completely explain the expression pattern of MGMT in clinical glioblastoma specimens. Here we used a genome-wide microarray-based approach to identify MGMT regulating miRNAs. Our screen unveiled three novel MGMT regulating miRNAs, miR-127-3p, miR-409-3p, and miR-124-3p, in addition to the previously identified miR-181d-5p. Transfection of these three novel miRNAs into the T98G glioblastoma cell line suppressed MGMT mRNA and protein expression. However, their MGMT- suppressive effects are 30–50% relative that seen with miR-181d-5p transfection. *In silico* analyses of The Cancer Genome Atlas (TCGA) and Chinese Glioma Genome Atlas (CGGA) revealed that miR-181d-5p is the only miRNA that consistently exhibited inverse correlation with MGMT mRNA expression. However, statistical models incorporating both miR-181d-5p and miR-409-3p expression better predict MGMT expression relative to models involving either miRNA alone. Our results confirmed miR-181d-5p as the key MGMT-regulating miRNA. Other MGMT regulating miRNAs, including the miR-409-3p identified in this report, modify the effect of miR-181d-5p on MGMT expression. MGMT expression is, thus, regulated by cooperative interaction between key MGMT-regulating miRNAs.

## INTRODUCTION

O^6^-methylguanine-DNA methyltransferase (MGMT) is an ubiquitously expressed nuclear enzyme which removes alkyl groups from the O^6^-position of O^6^-methylguanine (O^6−^MG) [1]. Each single alkyl group removed from O^6^-MG is transferred to a cysteine residue within the active site of MGMT, implying the inactivation of one molecule of MGMT enzyme for each alkyl group removed from methylguanine, a process termed suicide inhibition [2]. MGMT-mediated removal of alkyl groups from O^6^-MG is also relevant in alkylating chemotherapy with temozolomide and nitrosourea derivatives of gliomas, the most common malignant tumor of the brain. DNA alkylation produced by temozolomide causes base mispairing. The mismatched O^6^-MG to thymine base pair is recognized by the pathway involving the repair proteins MLH1, MSH2, MSH6 and PMS2, resulting in futile cycles of repair, leading to cell cycle arrest and cell death [3, 4]. The methylation damage produced by temozolomide can be reversed by MGMT, as its DNA repairing activity provides resistance against the cytotoxic effects of guanine methylation. As a consequence, patients affected by glioma that have been treated with the alkylating drug temozolomide show a better prognosis when MGMT expression is reduced because of promoter methylation [5], this observation has been confirmed by several subsequent studies [6–8]. Thus silencing the expression of MGMT gene is a relevant advantage for patients affected by glioma upon treatment with temozolomide, and a thorough understanding of the mechanisms of regulation of MGMT gene expression could provide useful hints for innovative therapeutic approaches.

MGMT expression is promoted by different nuclear transcription factors, namely NF-κB, Sp1, AP-1, CEBP, Hif-1α ανδ enhanced βy the acetylation of histones H3 and H4, as reviewed [9]. Down-modulation of MGMT expression is regulated by promoter methylation, although this mechanism does not always correlates with the levels of expression of MGMT transcript or protein [10, 11], thus suggesting the presence of further mechanisms of post-transcriptional regulation. MicroRNAs (miRNAs) (www.mirbase.org) belong to a family of small (19 to 25 nucleotides in length) noncoding RNAs that target specific mRNA, causing translational repression or mRNA degradation, depending on the degree of complementarities between miRNAs and the target sequences [12, 13]. By this mechanism of action miRNA are potent post-transcriptional regulators of gene expression. Although *in silico* analysis of the 3′-UTR of the MGMT gene has revealed several potential sequences that could be site for interaction of miRNAs [14], miRNA-dependent regulation of MGMT expression is presently under intensive investigation, but still not fully understood.

A genome-wide analysis of expression of 1,146 miRNAs performed in tissue samples obtained from glioblastoma specimens evidenced a role for miR-181d-5p expression as inversely correlated with a favourable prognosis. The favourable effect of miR-181d-5p was found related at least in part to its effect in down-modulating MGMT mRNA expression in A1207, LN340 and T98G glioblastoma cell lines [15]. Additional information has been provided by a bioinformatics analysis in the TCGA database related to glioblastoma [16], aimed to search inverse correlation between miRNAs levels and MGMT mRNA, taking into account also the contribution of the MGMT promoter methylation [17]. The bioinformatics analysis confirmed the role of miR-181d-5p and found miR-767-3p and miR-648 as novel potential regulators of MGMT gene expression [17]. Validation in experimental glioblastoma cell models *in vitro* supported the bioinformatics analyses and indicated that downregulation of MGMT expression by miR-181d-5p and miR-767-3p is due to degradation of the MGMT mRNA whereas miR-648 affects MGMT protein translation [17]. Further contribution indicated a role for the paralogues miR-221 and miR-222 [18], known to be highly expressed in glioblastoma tissues [19]. A fourth contribution was obtained by testing significant reduction of MGMT protein expression with a genome-wide miR screening performed by transfecting 885 known miRNAs into the T98G glioblastoma cell line, which indicated a regulatory role for miR-603, interacting directly with the 3′-UTR region of MGMT gene [20].

Considering that this series of miRNAs have been identified with different approaches and that the inverse correlation expected between MGMT expression and the expression of each miRNA is not usually characterized by very high and significant correlation coefficient, possibilities are open that further miRNAs will be revealed as regulators of MGMT expression and that the MGMT down-regulation might require the synergy of action of these and/or other miRNAs. Therefore, we decided to approach the search of further miRNAs potentially regulating MGMT gene expression by a novel approach in respect to the previous [15, 17, 18, 20]. Here we performed a genome-wide screening of 1,205 miRNAs in glioblastoma tissue samples with differential expression of MGMT mRNA in which MGMT gene promoter was non-methylated. We found inverse correlation between MGMT mRNA expression and the expression of several novel miRNAs. Validation of the candidate miRNAs in terms of quantitative expression and effect of MGMT modulation in glioblastoma cell line *in vitro* identified a novel role for miR-409 in the epigenetic regulation of expression of MGMT gene.

## RESULTS

### Identification of miRNAs inversely correlated with MGMT expression

**Figure 1:**
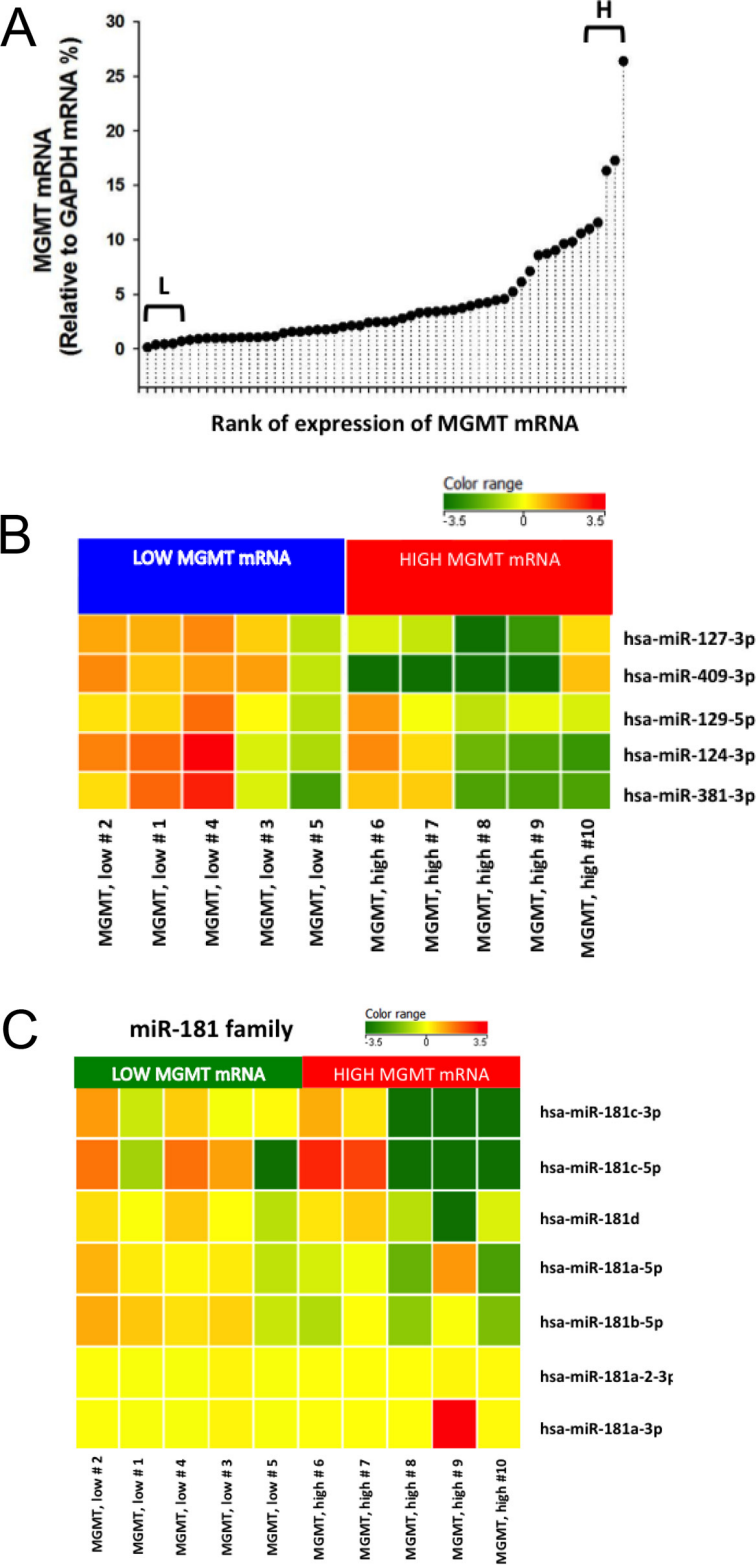
Initial screening of miRNAs related to MGMT expression in glioblastoma *ex vivo* MGMT mRNA was quantified in FineFix–fixed and paraffin-embedded 57 samples of glioblastoma (WHO grade IV). MGMT gene promoter was non-methylated in all samples. MGMT mRNA relative expression normalized over GAPDH mRNA was analyzed by RT-qPCR with TaqMan probes. (**A**) Relative expression was ordered in a rank in order to select samples at the extremes of low (L) and high (H) MGMT mRNA expression. (**B**) Expression pattern of five miRNAs differentially expressed between MGMT-low and MGMT-high glioblastoma samples (WHO grade IV), displaying extreme levels of MGMT mRNA. MGMT gene promoter was non-methylated in all samples. (**C**) MiR-181 family expression in the same low- and high-MGMT glioblastoma samples.

**Figure 2:**
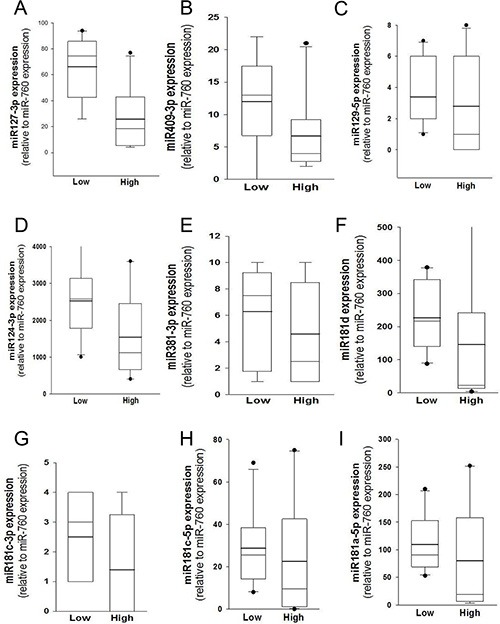
Validation of miRNA expression by RT-qPCR (**A**) Quantitative analyses of miR-127-3p, (**B**) miR-409-3p, (**C**) miR-129-5p, (**D**) miR-124-3p, (**E**) miR-381-3p, (**F**) miR-181d-5p, (**G**) miR-181c-3p, (**H**) miR-181c-5p and (**I**) miR-181a-5p. The two groups of extreme samples refer the specimens expressing to *low* (*n* = 5) and *high* (*n* = 5) MGMT mRNA. Analyses were performed with TaqMan probes and each miRNA was normalized to the most stable miRNA of the array (miR-760). Mean (bold line), median (light line), 5th and 95th confidence interval (box limits) and outliers as single dots are reported.

**Figure 3:**
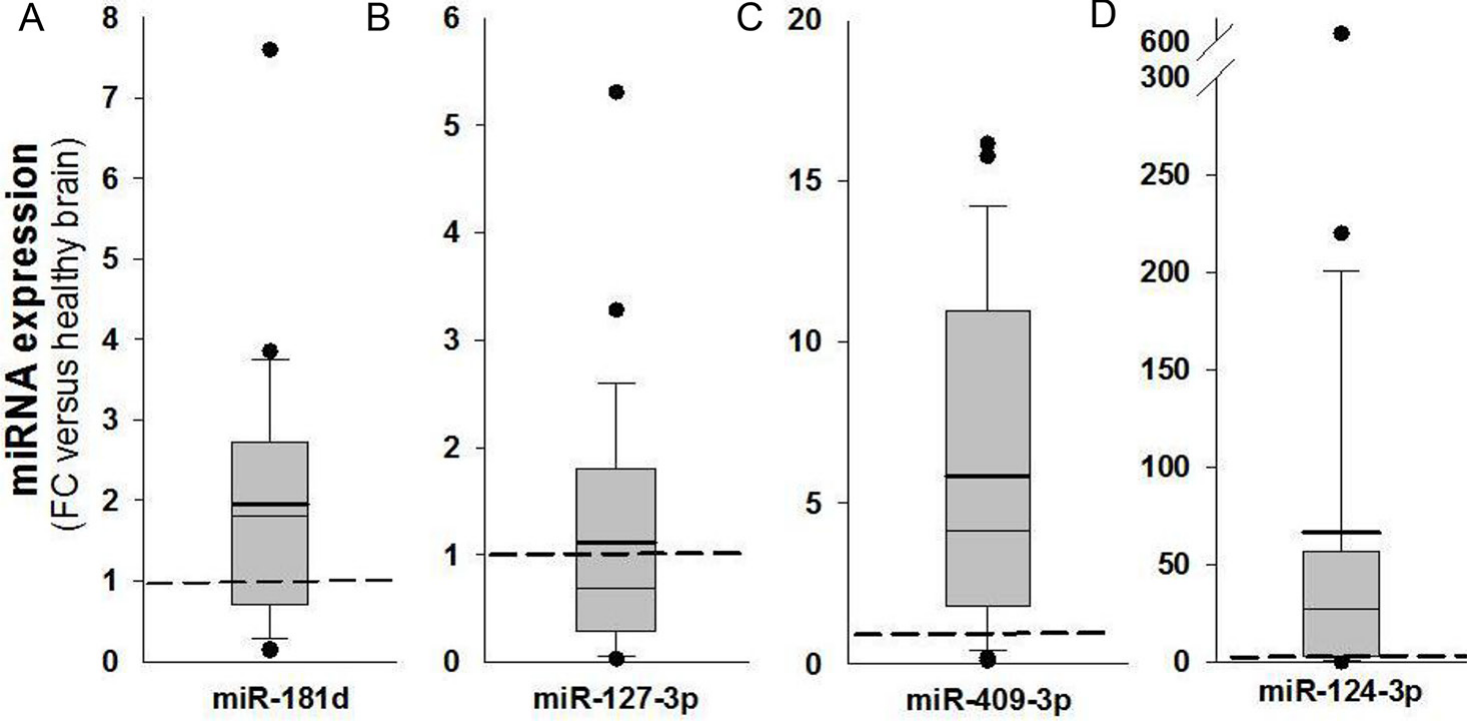
Analysis of expression of miR-181d-5p, miR-127-3p, miR-409-3p and miR-124-3p in glioblastoma tissues MiRNAs quantified by RT-qPCR in glioblastoma tissues (*n* = 26) are expressed as fold changes (FC) of a reference healthy brain RNA obtained from a commercial source (Clontech). Analyses were performed with TaqMan probes and each miRNA was calibrated to miR-U6. Dashed line represents the normalized level of expression in healthy reference RNA sample. Mean (bold line), median (light line), 5th and 95th confidence interval (box limits) and outliers are reported.

MGMT gene expression can be modulated by different epigenetic mechanisms, including promoter methylation and miRNA regulation [9]. MicroRNAs are known to regulate gene expression by translational repression or mRNA degradation [21]. We focused our investigation on miRNAs potentially intervening on MGMT RNA degradation, analyzing FFPE tissues from a cohort of 57 glioblastoma samples in which the MGMT promoter was non-methylated. Since the expression of MGMT and of its regulating miRNAs is expected to be inversely correlated, we quantified MGMT mRNA in all 57 glioblastoma samples (Figure 1A); then, we selected ten glioblastoma samples at the extremes in a rank of mRNA expression, as graphically reported in Figure 1A. Five samples with the lowest MGMT expression (Low MGMT mRNA) and 5 with the highest MGMT mRNA expression (High MGMT mRNA) in which the MGMT gene promoter was non-methylated were analyzed with Agilent miRNA microarray and compared. Based on microarray analysis, we identified 9 miRNAs differentially expressed between MGMT-low (L) and MGMT-high (H) tumors, reported in Supplementary Results (Supplementary Table S1). Five of these miRNAs, namely miR-127-3p, miR-409-3p, miR-129-5p, miR-124-3p, miR-381-3p, exhibited an average expression inversely correlated with that of MGMT mRNA, as graphically represented in Figure 1B. Two members of miR-181 family (miR-181b-5p and miR-181c-3p) were among the differentially expressed miRNAs (Supplementary Table S1). Since miR-181d-5p was previously reported as a regulator of MGMT expression [15], we examined the expression profile of miR-181 family members in MGMT-low and MGMT-high samples, as graphically represented in Figure 1C. An interesting inverse expression was indeed observed for miR 181c-3p, miR-181c-5p, miR-181d-5p and miR-181a-5p. The relative expression of the miRNAs identified from microarray analysis in the two groups of extremes in MGMT expression was further tested with RT-qPCR utilizing TaqMan probe assays. Each miRNA was normalized to miR-760, whose expression was found to be very stable in our microarray analysis. Validation of quantitative expression of miRNAs confirmed an inversely regulated pattern in relation to high and low MGMT mRNA for miRNAs 127-3p, 409-3p, 124-3p, 181d-5p, as shown in Figure 2A, 2B, 2D and 2F. In order to confirm in replicate samples the results obtained with the microarray analysis, the expression of these miRNAs was tested in a new set of tissue samples derived from glioblastoma. To facilitate the comparison of our results with those obtained by other researchers, each miR was normalized to the commonly utilized miR-U6, instead of the miR-760, and the expression relative to healthy brain RNA was carried on utilizing a commercial source, which could be easily available by other groups. As the expression of miRNAs 381-3p, 181c-3p, 181c-5p and 181a-5p did not show a strong inverse correlation with the expression of MGMT mRNA, as shown in Figure 2, panels 2C, 2E, 2G–2I, no further analyses were conducted on these miRNAs. As shown in Figure 3, the average expression levels of miR-127-3p and miR-181d-5p were similar to that of the reference RNA from healthy brain. Strikingly, the average expression of miR-409-3p was at least 5 folds the level found in healthy brain and that of miR-124-3p was even more expressed in glioblastoma than in healthy brain RNA, with an average 60 folds level. In all cases, the variability of the expression of the four miRNAs tested was remarkably high, as expected in glioblastoma specimens.

### Correlation of candidate miRNAs with MGMT expression

**Figure 4:**
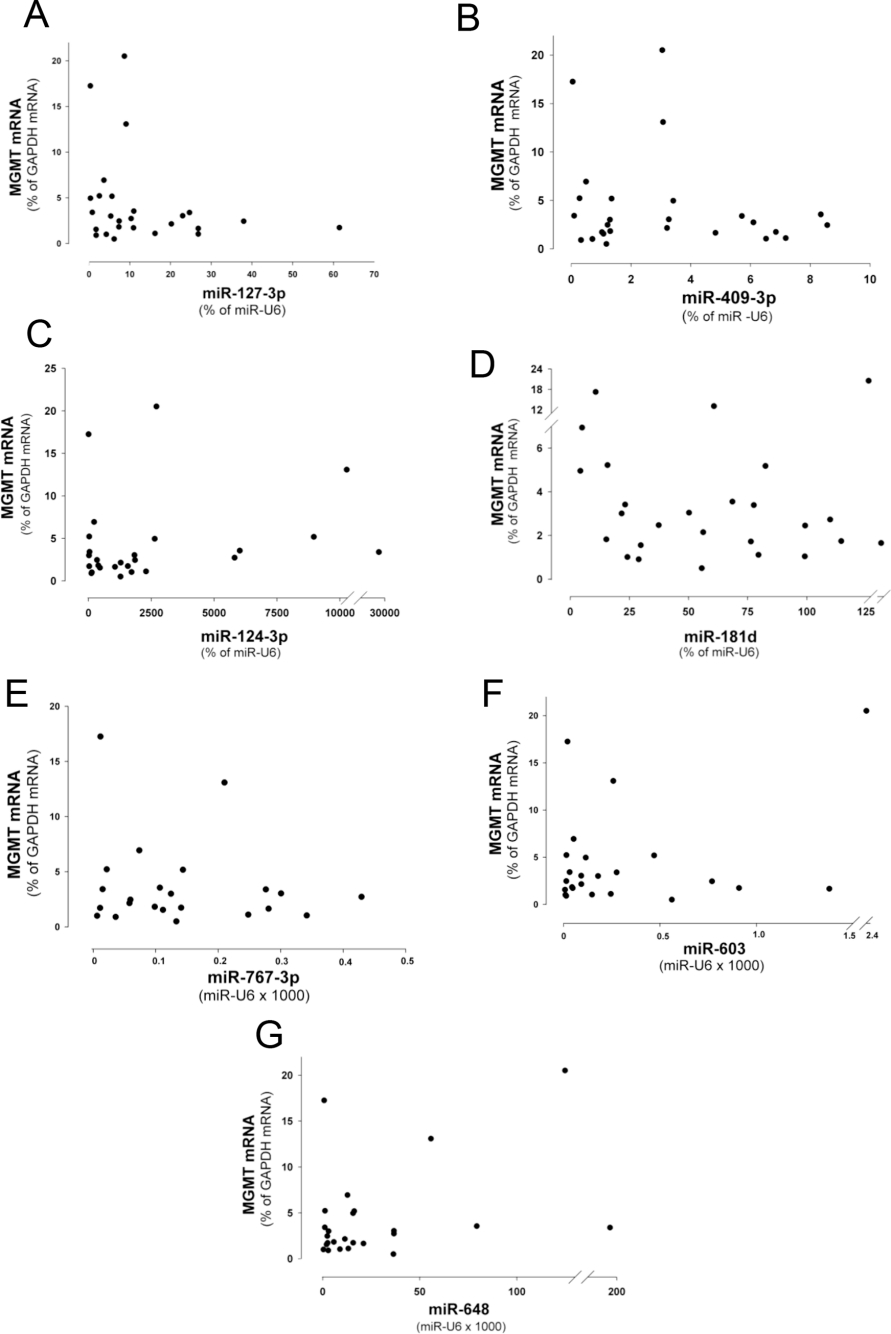
Scatter plots showing the relation between the levels of expression of MGMT mRNA and miRNAs (**A**) miR-127-3p, (**B**) -409-3p, (**C**) -124-3p, (**D**) -181d-5p, (**E**) -648, (**F**) -767-3p and (**G**) -603 in WHO 2007 grade IV glioblastoma with non-methylated MGMT promoter (*n* = 26).

After the screening performed by the miRNA profiling reported in Figure 1B and 1C, the expression of the miRNAs 127-3p, 409-3p, 124-3p, 181d-5p was expected to be inversely correlated with that of MGMT mRNA. Thus we plotted the levels of expression of the four miRNAs versus MGMT mRNA content in glioblastoma specimens in which the MGMT gene promoter was non-methylated. As shown in the dot plots reported in Figure 4A–4D, the four miRNAs show a general trend of inverse correlation with MGMT mRNA, although the correlation coefficients did not reach a statistical significance. Since miR-767-3p, miR-648 and miR-603, have been recently shown to down-modulate MGMT expression [17, 20], we verified their inverse correlation in the scatter plots reported in Figure 4E–4G. In analogy to the miRNAs analyzed previously, a similar trend of inverse correlation can be observed also for these miRNAs. Remarkably, also in this case we should underline that the correlation coefficient did not reach a statistic significance.

### Alteration of intracellular miRNAs to down-modulate MGMT expression

**Figure 5:**
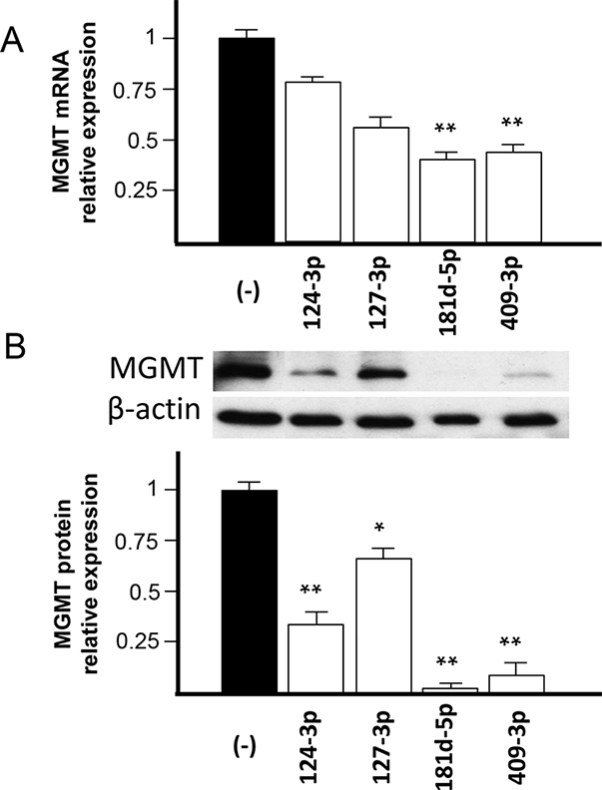
Effect of transfection of pre-miR-124-3p, -127-3p, -181d-5p and -409-3p on MGMT mRNA and protein expression in T98G glioblastoma cell line T98G glioblastoma cells were transfected with 200 nM of the pre-miRs indicated in the figure labels for each microRNA or scrambled sequence (−). (**A**) MGMT mRNA expression was detected with TaqMan probes, as indicated in the Method section, and the relative expression normalized to that of T98G cells transfected with scrambled sequence. (**B**) MGMT protein expression as detected by Western analysis is shown in the inset panel (using β-actin as reference). Band density was plotted and normalized to that of the T98G cells transfected with the scrambled sequence. Statistical significance in A and B at the levels of *p* < 0.05 (*) and *p* < 0.01 (**) is relative to cells transfected with scrambled sequence.

To verify the down-modulation of MGMT expression by increasing the intracellular concentration of the candidate miRNAs *in vitro*, we selected a glioblastoma cell line expressing costitutively a high level of MGMT mRNA. We checked MGMT mRNA expression in three glioblastoma cell lines, namely T98G, U251 and U373. As shown in Supplementary Table S2, T98G cell line presented a high MGMT mRNA expression level and it was chosen to test the effect of increasing the intracellular concentration of the candidate miRNAs. As shown in Figure 5A, transfection of the T98G glioblastoma cell line with pre-miR-124-3p, -127-3p, -181d-5p and -409-3p reduced the MGMT mRNA at different extents, the most significant down-modulation being obtained with the pre-miR-181d-5p and -409-3p. In parallel, we tested the expression of MGMT protein. As shown in Figure 5B, transfection with the pre-miR-181d-5p, -409-3p, -124-3p and -127-3p produced a significant down-modulation of expression of MGMT protein, with a particularly strong effect with pre-miRNAs-181d-5p and -409-3p.

### *In silico* analysis of candidate microRNAs in TCGA and CGGA datasets

The results reported in Figure 5 suggest that miR-181d-5p and -409-3p are the most potent in inhibiting MGMT expression causing both mRNA degradation and translational repression, whereas miR-124-3p and -127-3p have a modest effect on mRNA degradation and exert their inhibitory effect at translational repression. To gain further insights on the role of these microRNAs in glioblastoma we tested the impact of their expression levels on the overall survival of affected patients in The Cancer Genome Atlas (TCGA) for Glioblastomas and the Chinese Glioma Genome Atlas (CGGA) datasets. As shown in Supplementary Table S4, miR-181d-5p has a statistically significant impact on the survival of these patients, as observed in the two independent TCGA and CGGA datasets. In parallel, we tested the correlation between each candidate microRNA and the expression levels of MGMT mRNA in these datasets. Then we carried on a regression analysis reported in Supplementary Table S5 and plotted in Supplementary Figure S1. The analyses indicate that miR-181d-5p is significantly and consistently inversely related with MGMT mRNA in both TCGA and CGGA datasets (*p* value of 0.006 and 0.04, respectively), whereas the other microRNAs show a much weaker relation with MGMT mRNA, the best of the remaining three microRNAs being the miR-409-3p. In order to detect a possible interaction of these microRNAs in the down-regulation of expression of MGMT, we performed a multiple regression analysis of the miR-409-3p, -124-3p and -127-3p in respect to miR-181d-5p in association with MGMT mRNA levels. No significant interactions were found for miR-124-3p and -127-3p with miR-181d-5p in relation with MGMT mRNA expression. On the contrary, interaction between miR-181d and miR-409-3p versus MGMT mRNA was found statistically significant (*p* = 0.029). MiR-181d and MGMT mRNA values were plotted for miR-409-3 *p* values above (HIGH) or below (LOW) the median value of miR-409-3p, as shown in Supplementary Figure S2. Regression significance of MGMT mRNA with miR-181d for HIGH miR-409-3p was *p* = 0.017 (*) and for LOW miR-409-3p was *p* = 0.313, suggesting a potential positive interaction of miR-409-3p together with miR-181d-5p in down-modulating MGMT mRNA expression. In synthesis, here we confirm a pivotal role of miR-181d-5p in the down-modulation of MGMT expression, as previously reported [15], and identify a novel complementary role for miR-409-3p.

## DISCUSSION

Low level of MGMT expression in glioblastoma is associated with favourable response to the alkylating drug temozolomide in patients. The best estimate suggest that MGMT promoter methylation status accounts for ∼50% of variation in MGMT expression in clinical glioblastoma specimens (11). Recent studies suggest that miRNA regulation additionally contribute to the expression pattern of MGMT in clinical glioblastoma specimens. Here we performed a screen to identify novel MGMT-regulation miRNAs. We screened the expression of 1,205 miRNAs in glioblastoma tissue samples with differential expression of MGMT transcript. The top candidates were further tested for MGMT-suppressing activity and correlation with MGMT expression using the TCGA and CGGA glioblastoma databases. Our analysis confirmed a pivotal role of the previously published miR-181d-5p [15, 20] in MGMT regulation. Additionally, we demonstrate that MGMT expression is ultimately governed by cooperative interaction between miR-181d-5p and miR-409-3p.

We found that the expression level of miR-409-3p is 3/5-fold increased in glioblastoma specimens with respect to reference non-neoplastic brain tissue (Figure 3C). Although to the best of our knowledge it has not previously reported in glioma, expression of miR-409-3p has been found reduced in colorectal, gastric, bladder and ovarian cancers, suggesting a role as “tumor suppressor miR” [22–27]. Different important target genes have been validated in different cancers, such as the transcriptional regulator PHF10 [22], the pro-metastatic gene radixin [23], the pro-angiogenic gene angiogenin (ANG) [24], the oncoprotein GAB1 [27] and the pro-metastatic gene c-Met [25]. This considered, miR-409-3p has been proposed to cooperate in counteracting angiogenesis and metastatic processes in these cancers. An opposite role has been suggested in prostate cancer, where stromal fibroblasts deliver microvesicle-packaged miR-409-3p to epithelial prostate cells, which results in repressing tumor suppressor genes such as Ras repressor 1 and stromal antigen 2 [26]. Here we found an average 4-5 fold increase of expression of miR-409-3p in glioblastoma tissues *ex vivo*. Besides considering that increased expression of miR-409-3p in the glioblastoma cell line T98G strongly represses MGMT transcript and protein (Figure 5), which could favour a positive response to chemotherapy with alkylating agents in these patients, at the present time whether high miR-409-3p expression can be protective in relation to pro-angiogenetic and pro-metastatic processes in glioma is an interesting hypothesis that needs further investigation.

**Figure 6:**
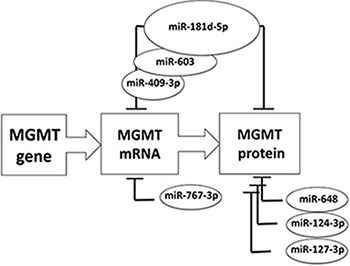
Schematic summary of major microRNAs reported to down-modulate MGMT expression MGMT expression is increased by different nuclear transcription factors (Sp1, NF-kB, AP-1, CEBP and HIF-1a), together with the acetylation of histones H3 and H4 and the stabilization by binding of N-myc Downstream Regulated Gene 1 (NDRG1) protein (for review see [31]). On the opposite, MGMT expression is downregulated by different mechanisms, namely methylation of the CpG islands in the promoter [32], di-methylation of histone H3K9 [33] and microRNAs. The graph represents the central role of miR-181d-5p [15] in down regulating MGMT expression, by acting both on MGMT mRNA degradation and interference with protein translation. Other and this report suggest that miR-603 [20], and 409-3p cooperate with miR-181d-5p in MGMT mRNA degradation, whereas miR-648 [17], miR-767-3p [17], -124-3p and 127-3p could play an ancillary role.

Transfection of the MGMT expressing T98G glioblastoma cells with the pre-miRNAs increasing the intracellular concentrations of miR-181d-5p, miR-409-3p, miR-124-3p and miR-127-3p produce reduced MGMT protein (Figure 5B). The MGMT suppressive effects were most dramatic with miR-181d-5p and miR-409-3p. These miRNAs are also the only candidates from our screen whose levels correlated with MGMT expression in the TCGA and CGGA glioblastoma database. The series of miRNAs proposed by previous [15, 17, 18, 20] and our work suggest a cooperative interaction of different miRNAs in MGMT-regulation the glioblastoma tissues *in vivo*. This frame work is largely consistent with proposed models of how microRNAs networks modulate glioblastoma biology [28]. Our working hypothesis is schematically represented in Figure 6. There are three lines of evidence that suggest miR-181d-5p as the key MGMT regulating miRNA. Of the reported MGMT-regulating miRNAs, miR-181d-5p 1) exert the most potent effect on MGMT expression – modulating both MGMT mRNA degradation and protein degradation 2) is the only MGMT-regulating miRNA that exhibit consistent inverse correlation with MGMT mRNA expression in multiple independent datasets (including the TCGA, CGGA, and the specimen collection), and 3) it is only MGMT-regulating miRNA that predict clinical survival in glioblastoma patients treated with TMZ [15]. In the context of the miRNA interactions observed in this study, we propose that MiR-409-3p, together with the previously identified miR-603 [20], cooperate with miR-181d-5p in MGMT regulation. Other miRNA, such as miR-124-3p, -127-3p, and others reported elsewhere [17], may exert an ancillary role in MGMT translation repression. All these microRNA-MGMT interactions thus deserve further investigation using 3′UTR luciferase assays, co-immunoprecipitation with biotinylated miRNAs and reversal of effect by anti-mirs, in parallel glioblastoma cell lines expressing at different levels MGMT and the miRNAs considered here.

## MATERIALS AND METHODS

### Human tissue samples

The analyses has been done retrospectically on human glioblastoma specimens, glioma grade IV according to WHO 2007 classification [29], obtained after surgery and fixed with the formalin-free alcoholic-based fixative FineFIX^®^ (Milestone SrL, Sorisole, Bergamo, Italy) and paraffin embedded, left over of samples previously utilized for histological diagnosis and in the archive of the Unit of Pathology. Histological diagnosis and grading has been confirmed separately by two expert pathologists (C.G. and A.E.) according to WHO 2007 classification [29]. Three 10 mm sections from each sample were utilized to extract RNA either for total RNA or miRNA analyses. Quantitative methylation of MGMT gene promoter was tested by pyrosequencing with MGMT plus^®^ reagents (Diatech Pharmacogenetics, Jesi AN Italy) according to the manufacturer's protocol. Analysis of expression of MGMT and candidate miRNAs was performed only in MGMT non-methylated samples. All procedures performed in these studies involving human samples were in accordance with the ethical standards of the institutional Ethical Committee of the University Hospital of Verona (CESC-VR RO) and with the 1964 Helsinki declaration and its later amendements or comparable ethical standards.

### RNA isolation

Total RNA to quantitate both MGMT mRNA and miRNAs was extracted from formalin-free alcoholic-based fixative FineFIX^®^ and paraffin embedded samples by MiRNeasy FFPE minikit (Qiagen, Venlo, Netherlands). Reference RNA from healthy brain was purchased from Clontech (Clontech Laboratories, Mountain View, CA, USA) and obtained from the whole brain of a 28-yr-old Asian male deceased because of sudden death. MicroRNA expression in glioblastoma and healthy brain RNA samples was firstly calculated relative to U6 snRNA. Samples from glioblastoma were subsequently expressed as Fold Changes (FC) in respect to reference RNA from healthy brain tissue. Total RNA from T98G cells was isolated using Tri-reagent^™^ (Sigma Aldrich). The 2100 bioanalyzer was used to determine the integrity and measure the concentration of total RNA samples (Agilent Technologies, Instrument DE54700480, Eukaryote Total RNA Nano Series II.xsy).

### Genome-wide miRNA screening

The global miRNA expression profile of low- and high-MGMT expressing glioblastoma was investigated using Agilent Human miRNA microarray (#G4870A, Agilent Technologies). This microarray consists of 60-mer DNA probes synthesized *in situ* and contains 60.000 features which represent 1200 human miRNAs, sourced from the Sanger miRBASE database (Release 16). RNA labeling and hybridization were performed following manufacturer's indications, as described. Agilent scanner and the Feature Extraction 10.5 software (Agilent Technologies) were used to obtain the microarray raw data. Microarray results were analyzed using the GeneSpring GX 13 software (Agilent Technologies). Data transformation was applied to set all the negative raw values at 1.0, followed by a quantile normalization and a log2 transformation. A filter on gene expression were used to keep only the miRNAs expressed in at least one sample. Differentially expressed genes were identified by applying a moderated *t*-test in with *p* < 0.05. Differentially expressed miRNAs were employed in Cluster Analysis, using the Manhattan correlation as a measure of similarity and the complete linkage rule for genes and samples clusterization. For cluster image generation, an additional step of normalization on gene median across all samples was added.

### Quantitation of MGMT mRNA content

Total RNA (1 μg) was reverse-transcribed to cDNA using the High Capacity cDNA Archive Kit and random primers (Applied Biosystems). RT-qPCR for MGMT mRNA was analyzed by TaqMan Gene Expression Assays (code Hs01037698_m1), and normalized to calibrator genes GAPDH mRNA (code Hs02758991_g1), according to the manufacturer's instructions, with a 7900HT Fast Real Time PCR System (Applied Biosystems). Relative quantification of gene expression was performed using the comparative threshold (C_T_) method as described by the manufacturer (Applied Biosystems User Bulletin 2).

### Quantitation of miRNAs

Quantitation of miRNAs was performed by specific reverse transcription and TaqMan probes with TaqMan MicroRNA Assays (Applied Biosystems, assay code 00232; 001182; 00229; 0011099). MiRNA expression was firstly normalized to miR-U6 (assay code 001973). Particularly, validation of miR-124 expression in glioblastoma tumors was performed by using a Taqman probe assay for hsa-miR-124-3p sequence that is a reverse complementary sequence compared to hsa-miR-124-5p as reported in Supplementary Table S3. MiRNA expression in glioblastoma and healthy brain RNA samples was firstly calculated relative to miR-U6 and subsequently expressed as Fold Change (FC) in respect to the reference healthy brain tissue.

### Glioma cell lines and culture conditions

T98G cells [30] were purchased from Sigma (Sigma-Aldrich, St. Louis, US) and were cultured in humidified atmosphere of 5% CO_2_/air in RPMI 1640 medium (Life Technologies, Monza, Italy) supplemented with 10% fetal bovine serum (FBS, Celbio, Milan, Italy), 100 U/ml penicillin and 10 mg/ml streptomycin (Sigma-Aldrich).

### Pre-miR transfections

T98G cells were seeded at 7 × 10^4^ cells/ml in 15 ml T75 flask and were transfected with 200 nM pre-miR-124-3p (PM10691), 127-3p (PM10400), 181d-5p (PM12522), 409-3p (PM12446), and the miR negative control (AM17110) (Ambion, Applied Biosystem, Foster City, CA, US) complexed with Lipofectamine RNAiMAX (Life Technologies, Carlsbad, CA, US). After 72 hours, total RNA was extracted and immediately converted to cDNA. Proteins were extracted in parallel for Western Blot analysis.

### T98G cell protein extract preparation

Treated or untreated T98G cells were washed three times with cold PBS and centrifuged at 1200 rpm for 10 min at 4°C. Then, cellular pellets were suspended in 50 μl cold water, frozen by dry ice for 5 min and vortexed for 10 s. This step was repeated 8 times consecutively, samples were finally centrifuged at 14000 rpm for 20 s and supernatant cytoplasmic fractions collected and immediately frozen at −80°C. The Pierce BCA Protein Assay Kit (Thermo Fisher Scientific, Waltham, MA, USA) was used to measure the protein concentration in the extract.

### Western blotting analysis

Twelve μg of cytoplasmic extracts were denatured for 5 min at 98°C in 1× SDS sample buffer (62.5 mM Tris-HCl pH 6.8, 2% SDS, 50 mM Dithiotreithol (DTT), 0.01% bromophenol blue, 10% glicerol) and loaded on SDS-PAGE gel (10 cm × 8 cm) in Tris-glycine Buffer (25 mM Tris, 192 mM glycine, 0.1% SDS). A biotinylated protein ladder (size range of 9–200 kDa) (Cell Signaling, Euroclone S.p.A., Pero, MI, Italy) was used as standard to determine molecular weight. The electrotransfer to 20 microns nitrocellulose membrane (Pierce, Euroclone S.p.A., Pero, Milano, Italy) was performed over-night at 360 mA and 4°C in electrotransfer buffer (25 mM Tris, 192 mM Glycine, 5% methanol). The membranes were prestained in Ponceau S Solution (Sigma Aldrich) to verify the transfer, washed with 25 ml TBS (10 mM Tris-HCl pH 7.4, 150 mM NaCl) for 10 min at room temperature and incubated in 25 ml of blocking buffer for 2 h at room temperature. The membranes were washed three times for 5 min each with 25 ml of TBS/T (TBS, 0.1% Tween-20) and incubated with MGMT primary mouse monoclonal antibody (1:1000) (Cat. GTX27045, GeneTex International Corporation, Alton Pkwy, CA, USA) in 15 ml primary antibody dilution buffer with gentle agitation over-night at 4°C. The day after, the membranes were washed three times for 5 min each with 20 ml of TBS/T and incubated in 15 ml of blocking buffer, in gentle agitation for 2 h at room temperature, with an appropriate HRP-conjugated secondary antibody (1:2000) and an HRP-conjugated anti-biotin antibody (1:1000) used to detect biotinylated protein marker. Finally, after three washes each with 20 ml of TBS/T for 5 min, the membranes were incubated with 10 ml LumiGLO^®^ (0.5 ml 20× LumiGLO^®^, 0.5 ml 20× Peroxide and 9.0 ml Milli-Q water) (Cell Signaling) in gentle agitation for 5 min at room temperature and exposed to x-ray film (Pierce). As necessary, after stripping procedure using the Restore™ Western Blot Stripping Buffer (Pierce) membranes were reprobed with primary and secondary antibodies. X-ray films for chemiluminescent blots were analyze by Gel Doc 2000 (Bio-Rad Laboratoires, MI, Italy) using Quantity One program to elaborate the intensity data of our specific target protein. The primary antibody against β-Actin (cat. 4970, Cell Signaling) was used as normalization control.

### Statistical analyses

Statistical significance was verified by Student's *t* test for unpaired data and reported only when observed at the significance levels of *p* < 0.05 (*) and *p* < 0.01 (**). Associations between MGMT mRNA levels, miR-181d-5p, miR-409-3p, miR-124-3p and miR-127-3p were evaluated by multiple linear regression models. Interactions between the four predictors were also investigated. Residual analysis was performed to assess the regression appropriateness. Statistical Package Stata version 12 (www.stata.com) was used for all statistical analysis.

## SUPPLEMENTARY MATERIAL TABLES AND FIGURES


