# Supplementary material for: miRNA array screening reveals cooperative MGMT-regulation between miR-181d-5p and miR-409-3p in glioblastoma
Source: Oncotarget. 2016 Apr 5;7(19):28195–206. doi: 10.18632/oncotarget.8618 (PMC5053720; doi:10.18632/oncotarget.8618)
Supplement: Supplementary file 1 [file oncotarget-07-28195-s001.pdf]

miRNA array screening reveals cooperative MGMT-regulation between miR-181d-5p and miR-409-3p in glioblastoma

Supplementary Materials

Supplementary Table S1: MicroRNAs differentially expressed between MGMT-low and MGMT-high glioblastomas

microRNA	*p*-value	Fold change	Regulation high/low	MGMT high (log2)	MGMT low (log2)
hsa-miR-124-3p	0.015034	16.0	down	3.80	7.80
hsa-miR-127-3p	0.041007	17.6	down	1.27	5.41
hsa-miR-129-5p	0.023617	2.7	down	3.24	4.65
hsa-miR-181b-5p	0.013845	2.6	down	6.16	7.53
hsa-miR-181c-3p	0.021823	19.1	down	−1.70	2.55
hsa-miR-381-3p	0.033114	11.1	down	−2.29	1.19
hsa-miR-409-3p	0.011395	47.9	down	−1.49	4.09
hsa-miR-4310	0.036081	13.6	up	0.83	−2.93
hsa-miR-616-3p	0.008522	8.9	up	1.05	−2.10

Supplementary Table S2: Expression of MGMT mRNA and candidate miRs in T98G, U251 and U373 glioblastoma cell lines

	Glioblastoma cell lines		
	T98G	U251	U373
miR-127-3p	0.85	0.09	0.31
miR-124-3p	9.72	2.24	8.69
miR-409-3p	0.2	0.04	0.11
miR-181d-5p	16.39	26.99	55.29
miR-767-3p	0	0	0
miR-648	0.03	0.02	0.06
MGMT mRNA	0.35	0.01	0.01

% expression relative to miR-U6 and GAPDH mRNA for microRNAs and MGMT mRNA, respectively.

Supplementary Table S3: Sequences of mature hsa-miR-124

microRNA	sequence	miRBase accession
hsa-miR-124-5p	CGUGUUCACAGCGGACCUUGAU	MIMAT0004591
hsa-miR-124-3p	UAAGGCACGCGGUGAAUGCC	MIMAT0000422

Sequence as reported in miRBase database (www.mirbase.org).

Supplementary Table S4: Survival analysis of candidate miRNAs in the TCGA and CGGA databases

	miR-181d-5p	miR-409-3p	miR-124-3p	miR-127-3p
**TCGA database**				
p value	0.003 (**)	0.467	0.997	0.577
**CGGA database**				
p value	0.004 (**)	0.668	0.575	0.885

TCGA database analysis from 199 patients affected by glioblastoma. CGGA database analysis from 82 patients affected by glioblastoma. Statistical significance at 0.05 (*) or 0.01 (**) levels, calculated as reported in Zhang et al. Neuro-Oncol. 2012; 14:712–9.

Supplementary Table S5: Regression analysis of candidate miRs versus MGMT mRNA in TCGA and CGGA databases

	miR-181d-5p	miR-409-3p	miR-124-3p	miR-127-3p
**TCGA database**				
r	−0.218	0.073	−0.011	0.025
p value	0.006 (**)	0.363	0.882	0.747
**CGGA database**				
r	−0.193	−0.008	−0.08	−0.032
p value	0.04 (*)	0.30	0.45	0.54

TCGA database analysis from 158 patients affected by glioblastoma. CGGA database analysis from 59 patients affected by glioblastoma. Two-tailed statistical significance at 0.05 (*) and 0.01 (**) is reported.

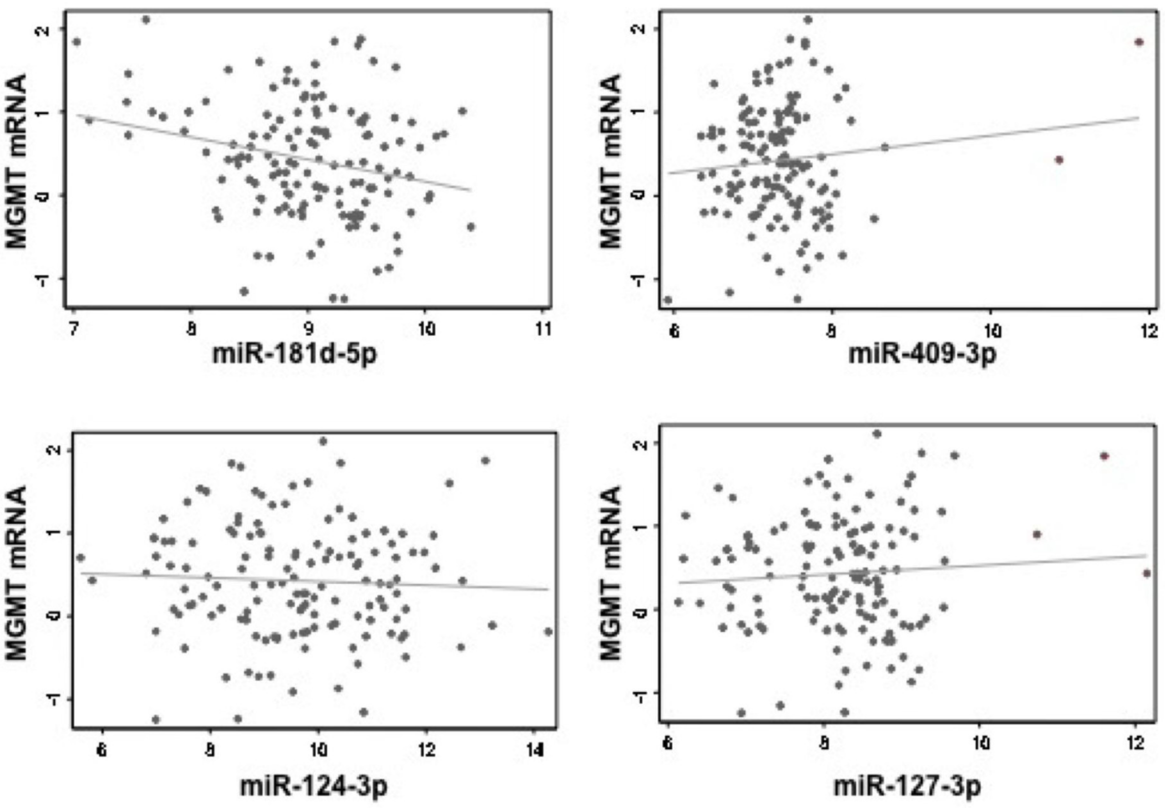

Supplementary Figure S1: Regression plots of miR-181d, -409-3p, 124-3p and 127-3p versus MGMT mRNA in glioblastoma samples from TCGA database. Regression values and statistical significance are reported in Supplementary Table S5.

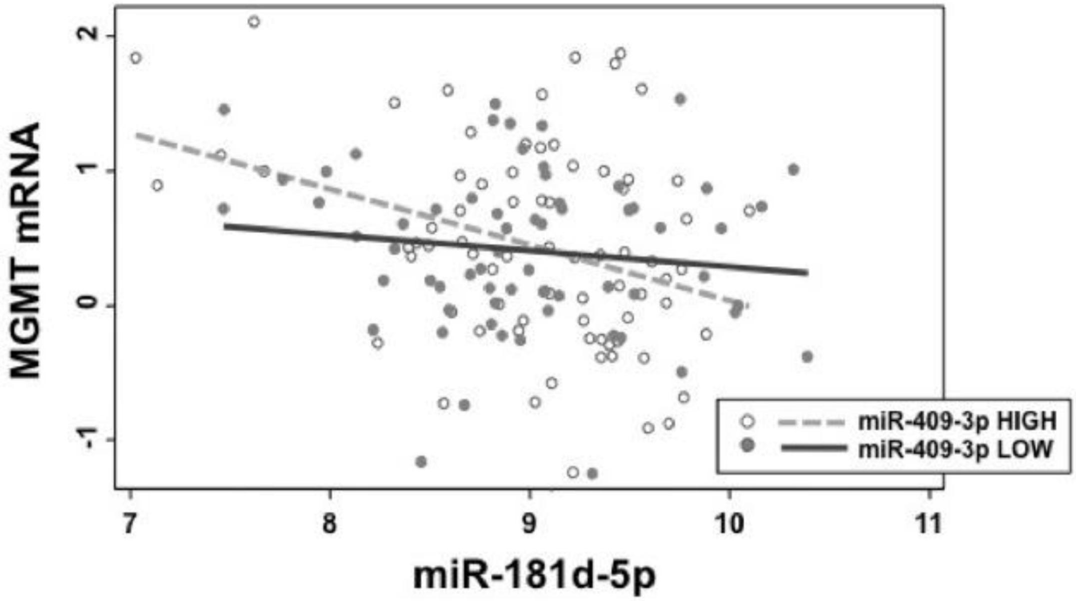

Supplementary Figure S2: Regression plot of miR-181d on MGMT mRNA as a function of high or low levels of miR-409-3p in glioblastoma samples from TCGA database. Interaction between miR-181d and miR-409-3p versus MGMT mRNA was found statistically significant ($p = 0.029$) as tested by multiple regression analysis. MiR-181d and MGMT mRNA values were plotted for miR-409-3p values above (HIGH) or below (LOW) the median value of miR-409-3p. Regression significance of MGMT mRNA with miR-181d for HIGH miR-409-3p was $p = 0.017$ (*) and for LOW miR-409-3p was $p = 0.313$.
